# Validation of the uncertainty stress scale-high-risk pregnancy - Chinese brief version: Rasch analysis

**DOI:** 10.1186/s12884-024-07078-7

**Published:** 2025-01-06

**Authors:** Yi Jing Tsai, Chi Chen Chen, Yu Yun Hsu, Chuan Chuan Chen

**Affiliations:** 1 Department of Nursing, College of Medicine, National Cheng Kung University, Tainan, Taiwan; 2 Research Center for Testing and Assessment, National Academy for Educational Research, New Taipei, Taiwan; 3 National Cheng Kung University Hospital, Tainan, Taiwan

**Keywords:** High-risk pregnancy, CFA, Rasch analysis, Uncertainty, Stress

## Abstract

**Background:**

High-risk pregnancy leads to uncertainty and stress in pregnant women due to the threatened of mother and fetus health. The Uncertainty Stress Scale High-Risk Pregnancy Version, a 54-item Chinese version (USS-HRPV-C), has been widely used to assess the uncertainty and stress that women experience during pregnancy. However, the length of the scale may result in a burden for respondents. Thus, a brief version of the USS-HRPV-C is needed for a concise and vigorous assessment. The aim of this study was to shorten the USS-HRPV-C and validate the brief version.

**Methods:**

This study used a cross-sectional design. A convenience sample of 200 women with high-risk pregnancies completed the 54-item USS-HRPV- C. Confirmatory factor analysis (CFA) and Rasch model to examine the construct validity of the short version of the USS-HRPV-C. Rasch analysis was used with a stepwise approach to select items with better goodness-of-fit and no differential item functioning (DIF). Additionally, Cronbachs’ α and Pearson correlations to evaluate the internal consistency of the original and brief versions. Test analysis modules (TAM) and Lavaan packages in R were used for data analyses.

**Results:**

The results of CFA supported a two-factor structure of the HRPV-C. Using the Rasch analysis, we reduced the USS-HRPV-C scale from 54 to 17 items. The selected 17 items were robust without displaying differential item functioning. Further, the 17-item short version exhibited satisfactory fit statistics that infit and outfit mean square ranged between 0.71 and 1.35, respectively. Internal consistency of Cronbach’s α for the short version of the USS-HRPV-C scale ranged was 0.90 and 0.92 for the subscales of uncertainty and stress respectively. Both subscales of the brief version were significantly related to the original version of USS-HRPV-C.

**Conclusions:**

This study developed a 17-item brief version of the USS-HRPV-C scale, which has demonstrated its satisfactory psychometric properties. Healthcare providers can use the validated brief version of the USS-HRPV-C to proficiently assess women’s psychosocial stress and uncertainty during pregnancy.

**Supplementary Information:**

The online version contains supplementary material available at 10.1186/s12884-024-07078-7.

## Background

The global prevalence of high-risk pregnancy is estimated to be between 22% and 25%, and the prevalence of high-risk pregnancy in Taiwan is around 13% [1]. The high-risk pregnancy not only threatens the health of mothers and the development of fetuses [2, 3] but also leads to pregnant women’s uncertainty and stress [4, 5].

Mishel [6] stated that uncertainty is a cognitive state wherein patients cannot determine events related to the disease. It can arise from unfamiliar events, unpredictable symptoms, lack of information, and inability to decide on treatment for disease [7]. On the other hand, stress arises from a stimulus that affects a person when the environment poses a threat. Stress refers to an individual’s feeling of being unable to bear a situation that exceeds their ability [8, 9].

When a pregnant mother is under stress, her body releases cortisol and other stress hormones, which can adversely affect maternal and fetal health [10]. Stress during pregnancy can affect fetal brain development, resulting in long-term developmental problems [11]. Stress resulting from uncertainty has been confirmed in previous studies to significantly affect emotions and health [12], leading to a corresponding decline in psychological well-being [13]. When individuals feel threatened by physical changes, they may find themselves facing a lot of uncertainty, which can lead to stress [14]. Therefore, assessing high-risk pregnant women’s uncertainty and stress levels is a critical prenatal responsibility [15].

The Uncertainty Stress Scale-High Risk Pregnancy Version (USS-HRPV) has been widely used to assess uncertainty and stressful status in women with high-risk pregnancies [16, 17]. The USS-HRPV was translated and tested into a Chinese version (i.e., USS-HRPV-C) [18, 19], a 54-item self-reported scale. The lengthy version of the USS-HRPV-C is needed to take substantial time to fill out, which may lead participants to respond arbitrarily to later items, potentially introducing measurement bias. Therefore, a brief version of the questionnaires has been recommended to improve respondents’ willingness [20]. Brief scales can achieve the accuracy of long-version original scales [21, 22].

However, appropriate psychometric testing methods should be implemented for developing a brief scale. Rasch analysis has been regarded as an efficient method to shorten scales by selecting the most robust and relevant items from the scale. Rasch analysis is based on item response theory to perform scale reduction measurement model [23]. Smith et al. (2010) used the latent trait model to estimate a person’s ability and item difficulty along a continuum. When paired with a Likert scale, the model can provide more information about the measurement’s psychometric features [24, 25]. This model can be used to objectively assess items and individual abilities [26, 27]. It enables the refinement of scales by removing items that do not fit the underlying measured dimensions, thereby advancing a revision of shorter scales [28]. The Rasch analysis model can be applied to shorten scales due to the following strengths: (1) conversion of raw scores, (2) separation of item difficulty and individual ability, and (3) testing item properties [29].

This study aimed to shorten and validate the USS-HRPV-C by selecting efficient items for women experiencing high-risk pregnancies. As such, this study used the Rasch model to shorten and revise the original USS-HRPV-C scale into a brief version. The Rasch model is based on three fundamental assumptions that ensure the validity and reliability of measurements. (a) unidimensionality: items must measure a single latent trait, ensuring the construct is coherent and not influenced by other factors. (b) local independence: item responses should be statistically independent, given the latent trait, with no influence from responses to other items. (c) non-speeded test: the test should measure latent ability, not response speed, requiring sufficient time for all items to be answered without time pressure [30–32]. These assumptions form the foundation of the Rasch model, guiding the development and evaluation of psychometric instruments. By meeting these criteria, the Rasch model ensures the precision and validity of scale-based measurements.

Given the aims of this study, we first used Principal Component Analysis (PCA) to examine the scale’s eigenvalues. We confirmed that the USS-HRPV scale is one factor that aligns with the study data’s essentially one-dimensional nature [33]. Second, we conducted a Confirmatory Factor Analysis (CFA) to assess whether the data supported a one-factor structure. Therefore, if the study data meets the criterion of unidimensionality, Rasch analysis can be conducted. Additionally, we examined inter-item correlations to ensure they were not excessively high, thereby avoiding multicollinearity and satisfying the Rasch model’s assumption of local independence.

## Methods

### Design, setting, and participants

This study was a cross-sectional design to test the USS-HRPV-C and shorten its scale using Rasch analysis. Participants were recruited from the obstetrics and gynecology clinic of the medical hospital in southern Taiwan. Subjects were at least 20 years old and diagnosed with a high-risk pregnancy between 20 and 40 weeks of gestation, and could read, write, and converse in Chinese. Women with mental disorders or intrauterine fetal death (IUFD) were excluded from participating in the study. Prior to shortening the USS-HRPV-C, the factor structure of the original USS-HRPV-C was verified using the confirmatory factor analysis (CFA), which ensures the appropriateness of the USS-HRPV-C to develop a shortened version.

After recruiting 223 high-risk pregnant women, only 200 provided completed data and participated in the study. Twenty-three women were excluded due to hospitalization for disease treatment, including preterm birth (*n* = 6), gestational diabetes mellitus (*n* = 5), hypertensive disorders of pregnancy (*n* = 8), and intrauterine fetal death (*n* = 4).

The mean age of the participants was 34.4 years (SD = 4.22, range = 22– 44 years old), with a mean gestation of 30 weeks (SD = 4.9, range = 20–39 weeks). Over half of the participants (55.5%, *n* = 111) were primiparous women, and over three-quarters (77.5%, *n* = 155) had a university education. Nearly all participants were married (95.5%, *n* = 191). High-risk pregnancy was diagnosed as 22.5% early uterine contraction (*n* = 45), 19% gestational diabetes mellitus (GDM) (*n* = 38), 13% hypertensive disorders of pregnancy (HDP) (*n* = 26), and 10.5% antepartum hemorrhage (APH) (*n* = 21).

### Instruments

#### Uncertainty Stress Scale High Risk-PregnancyVersion (USS-HRPV)

Clauson [34] developed the USS-HRPV to measure the degree of uncertainty and the resulting stress, threat, and feeling in high-risk pregnancy circumstances. The original scale contains 56 items with two subscales: uncertainty (29 items) and stress (25 items). Chen and Chen (2000) translated the USS-HRPV into a Chinese version (USS-HRPV-C) using Brislin’s (1986) [35] translation model. During the translation and verification of the Chinese version, two items were deleted due to cultural diversity concerns, resulting in a 54-item scale.

The degree of uncertainty of high-risk pregnancy-related diseases was assessed using a 5-point Likert scale (1 = definite, 2 = 25% uncertainty, 3 = 50% uncertainty, 4 = 75% uncertainty, 5 = 100% uncertainty). The total uncertainty score ranges from 54 to 270, with higher scores indicating higher uncertainty. The same items were also used to evaluate stress levels with a 3-point Likert scale (1 = no stress, 2 = some stress, 3 = extreme stress). Total stress score ranges from 54 to 162, with higher scores indicating higher stress levels resulting from uncertainty. At the end of the scale, four Visual Analogue Scales (VAS) ranging 0-100 points are used to assess the overall uncertainty, stress, threat, and feelings (0 = no uncertainty, no stress, no threat, and no positive feelings, and 100 = very high uncertainty, very high stress, very high threat, and very high positive feelings).

The USS-HRPV-C has had satisfied psychometric propterties. The scale showed satisfactory internal consistency, with a 0.96 Cronbach’s alpha and acceptable convergent validity. The uncertainty subscale score positively correlated with the overall VAS uncertainty (*r* = 0.47, *p* < 0.01). The stress subscale positively correlated with overall VAS stress (*r* = 0.65, *p* < 0.01) [18].

### Data collection

First, two of the authors and one research assistant collected data from the antenatal outpatient department (OPD) of a medical center in Taiwan. The researchers approached pregnant women in the antenatal care OPD to explain the purpose of the study. If the pregnant women met the inclusion criteria and were willing to participate in the study, written informed consents were obtained from the participants before data collection. Then, the researchers provided detailed instructions to ensure that participants understood and could complete the questionnaire. All data were collected anonymously and in a private setting. Participants completed the 54-item USS-HRPV-C and the demographic information after their prenatal care visit. Data collection occurred from September 2020 to March 2021. All data were transcribed into an Excel database and then coded for further analysis in R.

### Data screening before analyses

**Fig. 1:**
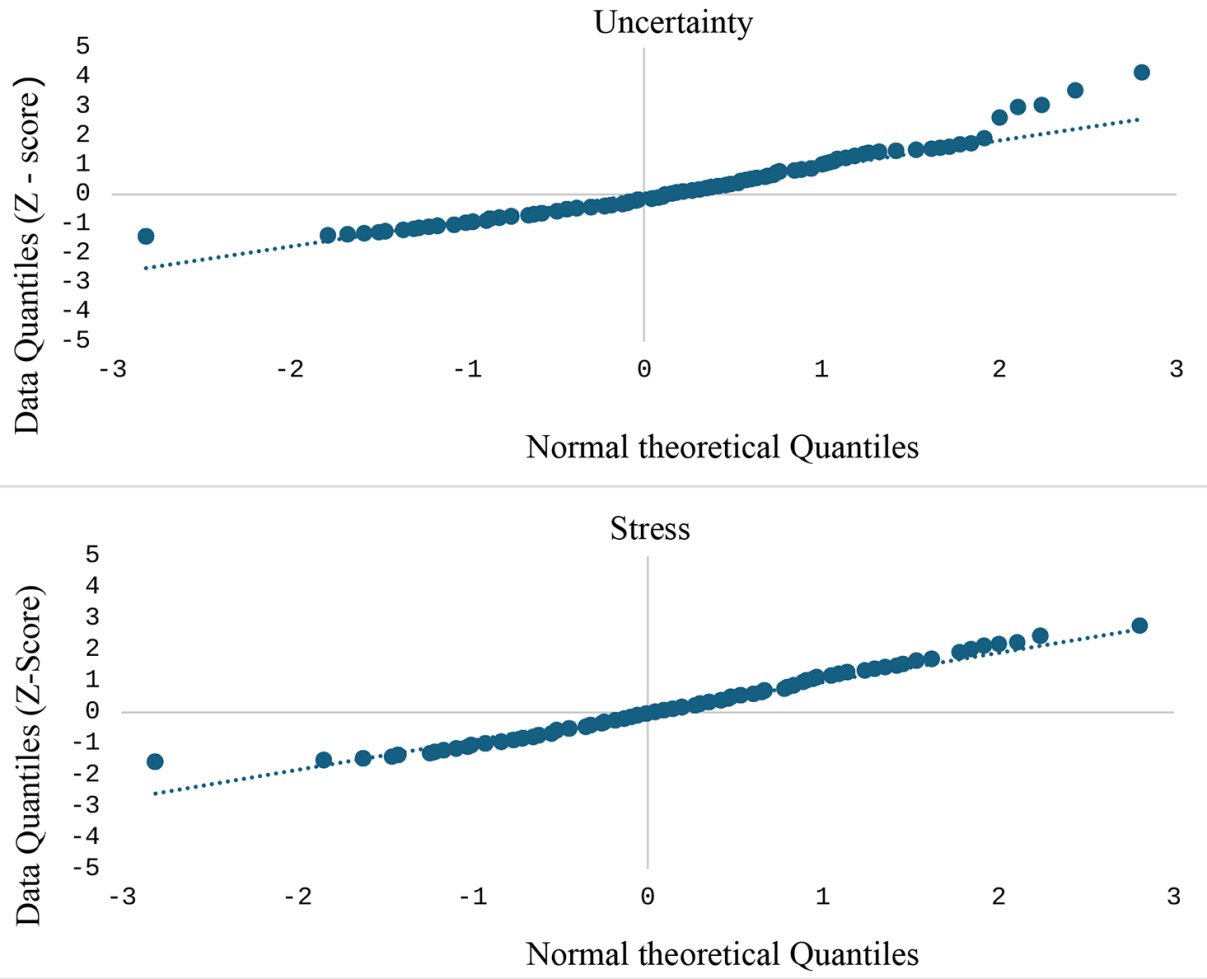
QQ plot of uncertainty and stress subscales

Before the data analyses, we conducted comprehensive data screening to ensure the quality and integrity of the dataset. Missing data were minimal (< 5%) and were addressed using mean substitution to maintain the sample size of 200 participants [36, 37]. While potentially small, our sample size aligns with established CFA guidelines [38, 39]. We assessed the normality of item responses and examined skewness and kurtosis statistics. Although some items exhibited slight deviations from normality, the overall impact was considered negligible due to the sufficient sample size [40]. The Quantile-Quantile plot (QQ plot) of uncertainty and stress further confirmed a normal distribution (Fig. 1). Before performing CFA and Rasch analysis, we evaluated the scale’s internal consistency using Cronbach’s alpha coefficients and item-total correlations. This step ensured that the items reliably measured the constructs of USS-HRPV.

### Data analysis

We used The Test Analysis Modules (TAM) and the latent variable analysis (lavaan) packages in the R software V.4.0.2 (R Foundation for Statistical Computing, Vienna, Austria) for the data analysis. Specifically, this study conducted CFA using the lavaan [41], and the Rasch analysis was conducted using the TAM [42].

This study used four-stage guidelines to select items from the original long version to a brief version: (1) Using CFA to confirm the structure of the USS-HRPV-C, (2) Conducting a Rasch analysis and differential item functioning (DIF) assessment to determine problematic items and item equivalency, respectively, (3) Hosting an expert panel discussion to determine the final brief version, (4) Conducting a CFA to confirm the construct and concurrent validity of the brief version of USS-HRPV-C [23, 29]. After that, we compared the differences between the original and the brief versions in various psychometric properties, such as item difficulty, the goodness of fit, and reliability, to evaluate the appropriateness of the shortened version of the USS-HRPV-C scale.

### Confirmatory factor analysis

We applied the CFA using the Diagonally Weighted Least Squares (DWLS) estimation method and a unifactorial model to verify that the dimensions of the USS-HRPV-C scale and the structures of the brief version are in line with the original structures [43]. Given the ordinal nature of the USS-HRPV-C scale, which uses Likert-type responses, DWLS is more appropriate for ordinal data and provides more accurate parameter estimates under these conditions [44, 45]. Indicators that the model was a good fit for the data were as follows: A normed *x*^2^ below 3.0, comparative fit index (CFI) above 0.9, non-normed fit index (NNFI) above, also known as Tucker-Lewis Index (TLI) above 0.9, root-mean-squared error of approximation (RMSEA) values below 0.06, and a standardized root-mean-squared residual (SRMR) below 0.08 suggest acceptable fit [46]. We performed a principal component analysis (PCA) on raw data to evaluate its eigenvalues. The scree plot (Additional file 1) indicates that the uncertainty and stress subscales conform to a one-factor structure. This finding demonstrates essential unidimensionality, which aligns with the unidimensionality assumption of the Rasch model [47, 48], thereby supporting the application of Rasch analysis.

### Rasch model

Item response theory involves fitting a scale to a specific model. A scale can be reduced by comparing the original and short-form scale estimates. We used the Wright map to evaluate the distribution of item difficulty and personal ability, considering that items threshold onto the same scale as a latent feature. We used the partial credit model (PCM) instead of the rating scale model (RSM) because our items had varying response categories. The PCM provides greater flexibility by allowing each item to have its response structure, while the RSM requires identical response categories for all items, which was not suitable for our scale [49]. The PCM can be applied to the polytomous scaling model [50].

The mean square (MNSQ) was used to evaluate the items fit and is the most common statistics tests (goodness of fit), including two types of infit (weighted mean square), and outfit (unweighted mean square) [51]. While the value of MNSQ is between 0.5 and 1.5, it indicates a good data fit. If the infit or outfit MNSQ values are out of range, the items would be diagnosed as likely not fitting the Rasch model conditions and they may be considered for deletion from the scale [52]. Next, we evaluated item difficulty, which in Rasch analysis refers to the relative ease or difficulty with which respondents endorse an item. Although Likert-type items are ordinal, Rasch modeling allows us to place items on a latent continuum of difficulty. We employed a sequential process to refine the scale, balancing psychometric properties to enhance measurement precision. Item difficulty was determined using the item information function, which quantifies how much information an item contributes to measuring the latent construct at varying respondent ability levels. Items at extreme difficulty levels (very easy or difficult ) typically provide limited information, as their measurement precision diminishes when respondent abilities fall far from the item’s difficulty level [53]. Following established psychometric guidelines, such items were considered for deletion. This approach aimed to optimize the scale’s psychometric properties of the brief version.

### Differential item functioning (DIF) assessment

In the evaluation of item quality, attention must be given not only to item difficulty and discrimination but also to the fairness of the items. DIF assessment is crucial in ensuring fairness and validity in tests because it identifies items that may function differently for diverse subgroups, potentially leading to biased conclusions. DIF refers to a situation in which individuals from different groups, despite having the same ability level, have differing probabilities of answering an item correctly [54]. DIF assessment research can be based on different theoretical frameworks. First, response patterns are modeled using contingency tables or regression methods, such as the Mantel–Haenszel method [55], and the Logistic Regression method [56]. Second, based on IRT, the likelihood ratio test [57] is used to compare differences between two models across different groups. Third, DIF is considered a simultaneous item bias test (SIBTEST) arising from different dimensional abilities, employing the SIBTEST method [58]. Fourth, Similar to the multidimensional ability approach, DIF is viewed as a factor influencing responses. The significance of this influence is examined using the multiple indicators and multiple causes (MIMIC) method [54]. Various DIF detection methods can provide satisfactory test performance. However, when a test contains a large number of DIF items, it is necessary to employ different strategies to reduce the inflation of Type I errors. Strategies commonly used in previous studies include scale purification (SP) and DIF-Free-Then-DIF (DFTD) [59]. In this study, considering the structure of the scale, we utilized confirmatory factor analysis (CFA) based on structural equation modeling (SEM). To ensure consistency of the statistical model, the DIF detection method employed an SEM-based multiple-indicators multiple-causes (MIMIC) model in conjunction with the DFTD strategy. To ensure item equivalency and to determine whether item difficulty varied among primiparous and multiparous women [54].

### Panel discussion

After reducing items from the original scale using Rasch analysis, we conducted an expert panel discussion to determine the final brief version of USS-HRPV-C. The panel included five experts who have expertise in high-risk pregnancy care, obstetric nursing, or measurement properties. All panel experts participated in the content validity evaluation. The panel discussion guideline simultaneously considered the literature knowledge and psychometric statistics (e.g., removing items with high floor and ceiling effects and retaining the easiest and most difficult items to increase the scale`s sensitivity).

### Validation and reliability of the brief version of the USS-HRPV-C

We again applied the CFA to confirm the structure of the brief version. Additionally, we used Pearson’s correlation coefficient to examine the criterion-related validity of the brief USS-HRPV-C with the original version. We examined convergent validity via the correlations between the items in each factor and their corresponding VAS scores. The composite reliability (CR) and average variance extracted (AVE) were used as indicators for measuring the convergent validity of the brief version of USS-HRPV-C. When AVE ≥ 0.5 and CR ≥ 0.6 were considered adequate to verify the consistency of the psychological measurement between the original and brief versions [46].

## Results

### Descriptive analyses of items

The skewness values ranged from − 0.04 to 3.20. The kurtosis values varied from − 1.00 to 11.23. A slightly negative skewness indicates that respondents tended to select higher scores on an item, while a positive skewness suggests a tendency toward lower scores. The correlation matrix between items (Additional file 2).

### Confirmatory factor analysis(CFA)

CFA supported two distinct single-factor structures: uncertainty and stress. Due to their conceptual independence, each construct was analyzed separately to align with the study’s focus on these two dimensions. The fit indices for the uncertainty and stress results were 1.33–2.13 for χ^2^/df, 0.980–0.983 for NNFI, 0.980 − 0.983 for CFI, 0.041–0.075 for the RMSEA, and 0.089–0.096 for the SRMR. Based on the analysis results and path diagrams (Additional file 3), the single-factor models for uncertainty and stress demonstrated a good fit. These findings were consistent with the original scale study, indicating an acceptable model fit for both single-factor structures in the CFA analyses.

### Wright map

**Fig. 2:**
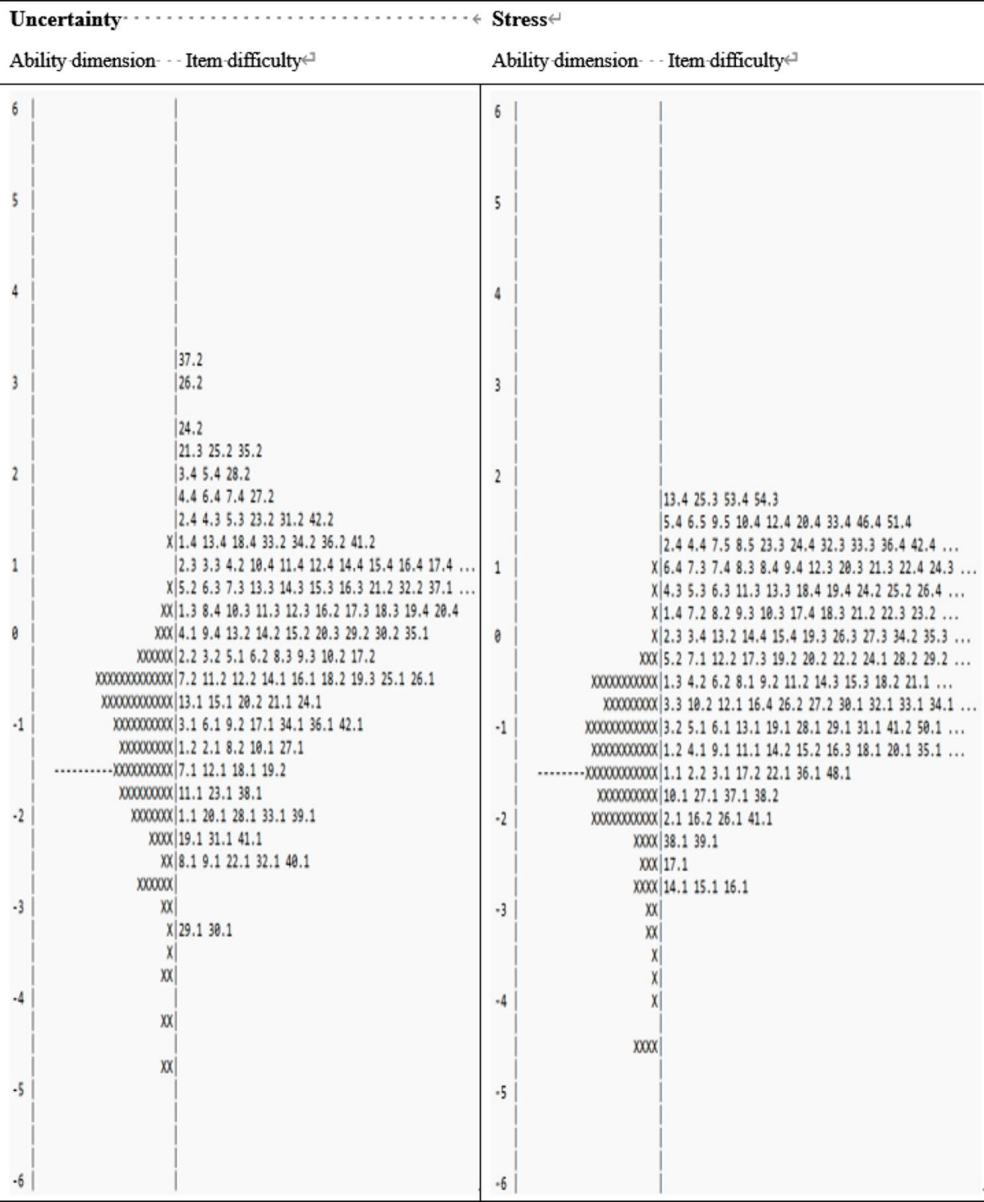
Wright map of distribution for uncertainty and stress subscales. *Note.* Each ‘X’ represents 2 subjects on the left. The number represents each item difficulty on the right, the number decimal point represents the threshold of the item

The Wright map (Fig. 2) shows the subscales of uncertainty and the distribution of a person’s abilities and item difficulty. The left-hand column represents a person’s ability, where that person with higher ability is displayed at the top of the figure, and those with lower ability at the bottom. The 54-item uncertainty difficulty thresholds ranged from − 3 to 3.3 logits, while the personal measures ranged from − 4.8 to + 3.5. The stress difficulty thresholds for the 54-item scale ranged from − 2.9 to 1.9 logits. In comparison, person measures ranged from − 4.5 to + 1 logits, indicating that the 54-item scale has the most difficult and easiest distribution items.

### Rasch analysis

During the process of infit and outfit iteration, seven items were removed from the original 54-item version due to their infit statistics falling below the expected model standards. The MNSQ revealed the infit and outfit data in the subscale uncertainty was 0.73 and 3.32, respectively; the infit and outfit data in the stress subscale was 0.76 and 1.69, respectively. Item ease and difficulty were assessed for item reduction. As illustrated in step 2 of Tables 1 and 49 items were deleted from the uncertainty subscale, and 43 items were deleted from the stress subscale.

**Table 1:** Selection of items of the USS-HRPV-C to a brief version

	Uncertainty								Stress								Final
Item	Step 1			Step2		Step3	Step4	Step5	Step 1			Step2		Step3	Step4	Step5	Brief Version Item
	Infit MNSQ	Outfit MNSQ	Item removal based on MNSQ	Difficulty distribution (SE)	Item removal based on difficult	Item removal based on DIF	Panel discussion retained item	Brief Version Result	Infit MNSQ	Outfit MNSQ	Item removal based on MNSQ	Difficulty distribution (SE)	Item removal based on difficult	Item removal based on DIF	Panel discussion retained item	Brief Version Result	
1	2.1	3.32	Removed	1.52 (.29) ^‡^	Removed	anchor item			1.44	1.69	Removed	1.34 (.27)		anchor item			
2	1.27	1.32		1.19 (.32)				Yes	1.13	1.18		0.33 (.26) ^†^	Removed				Item − 1
3	1.78	2.7	Removed	1.48 (.28)		✔			1.16	1.16		1.40 (.26)		✔			
4	0.91	0.81		2.69 (.38) ^‡^	Removed				0.93	0.84		0.82 (.27)				Yes	Item − 2
5	0.84	0.72		3.40 (.49) ^‡^	Removed				0.94	0.97		0.71 (.28)				Yes	Item − 3
6	1.07	1.34		3.15 (.43) ^‡^	Removed				0.88	0.78		3.07 (.37) ^‡^	Removed				
7	1.04	0.89		4.19 (.68) ^‡^	Removed	anchor item			1.22	1.33		3.17 (.35) ^‡^	Removed				
8	0.9	0.8		4.35 (.61) ^‡^	Removed				1.06	1.2		3.67 (.47) ^‡^	Removed				
9	1.03	1.1		2.82 (.39) ^‡^	Removed				1.01	0.91		1.89 (.29) ^‡^	Removed				
10	1.06	1.05		2.19 (.37) ^‡^	Removed				0.95	0.88		0.96 (.30)				Yes	Item − 4
11	0.88	0.77		1.40 (.21) ^‡^				Yes	0.95	0.87		1.12 (.27)				Yes	Item − 5
12	0.88	0.7		5.35 (.57) ^‡^	Removed				0.94	0.77		3.26 (.33) ^‡^	Removed				
13	0.9	0.72		4.74 (.53) ^‡^	Removed				1	0.94		2.75 (.32) ^‡^	Removed				
14	1.08	1.03		0.24 (.30) ^†^	Removed		Keep		1.18	1.19		1.84 (.28) ^‡^	Removed		Keep		Item − 6
15	1.13	1.08		0.33 (.30) ^†^	Removed		Keep		1.05	1.03		1.97 (.29) ^‡^	Removed		Keep		Item − 7
16	1.66	1.81	Removed	-0.48 (.30)^†^	Removed				1.38	1.31		0.03 (.25) ^†^	Removed				
17	1	0.95		0.34 (.30) ^†^	Removed				0.76	0.73		0.52 (.27)				Yes	Item − 8
18	1.27	1.68	Removed	2.50 (.34) ^‡^	Removed				1.08	1.08		0.39 (.27) ^†^	Removed				
19	1.02	0.94		4.07 (.39) ^‡^	Removed				0.83	0.71		2.45 (.31) ^‡^	Removed				
20	0.82	0.8		4.11 (.47) ^‡^	Removed				0.92	0.85		1.06 (.31)				Yes	Item − 9
21	0.73	0.56		3.05 (.28) ^‡^	Removed				0.85	0.69		3.21 (.41) ^‡^	Removed				
22	1.11	1.4		2.71 (.38) ^‡^	Removed				0.95	0.87		1.04 (.28)				Yes	Item − 10
23	0.82	0.76		2.41 (.28) ^‡^	Removed				0.86	0.71		3.18 (.46) ^‡^	Removed				
24	0.87	0.67		6.45 (.73) ^‡^	Removed	anchor item			0.9	0.75		3.84 (.42) ^‡^	Removed				
25	0.83	0.64		3.60 (.42) ^‡^	Removed				0.93	0.66		5.14 (.63) ^‡^	Removed	anchor item			
26	0.98	0.93		1.63 (.32) ^‡^	Removed		Keep		1.14	1.08		0.33 (.25) ^†^	Removed	✔	Keep		Item − 11
27	0.93	0.88		1.85 (.32) ^‡^	Removed		Keep		1.31	1.35		0.45 (.26) ^†^	Removed		Keep		Item − 12
28	0.99	0.89		4.56 (.42) ^‡^	Removed				0.94	0.84		2.30 (.31) ^‡^	Removed				
29	1.21	1.17		4.54 (.43) ^‡^	Removed	✔			1.23	1.58	Removed	2.27 (.28) ^‡^	Removed	✔			
30	1.46	1.61	Removed	3.38 (.38) ^‡^	Removed				1.07	0.98		3.76 (.39) ^‡^	Removed				
31	1.38	1.51	Removed	3.35 (.39) ^‡^	Removed				1.2	1.31		2.04 (.27) ^‡^	Removed				
32	0.94	0.85		2.99 (.29) ^‡^	Removed				0.89	0.87		2.93 (.36) ^‡^	Removed				
33	0.79	0.66		5.59 (.63) ^‡^	Removed				0.85	0.7		3.32 (.38) ^‡^	Removed				
34	1.26	1.43		4.97 (.43) ^‡^	Removed				1.11	0.85		5.50 (.64) ^‡^	Removed	✔			
35	1.04	0.91		2.62 (.33) ^‡^	Removed				1	1.05		0.71 (.25)				Yes	Item − 13
36	0.84	0.74		2.29 (.35) ^‡^	Removed				0.83	0.76		0.41 (.26) ^†^	Removed				
37	0.91	0.82		1.89 (.33) ^‡^	Removed		Keep		0.96	0.89		0.32 (.26) ^†^	Removed		Keep		Item − 14
38	1	1.08		0.29 (.29) ^†^	Removed				1	0.96		0.40 (.26) ^†^	Removed				
39	0.88	0.82		0.95 (.30)				Yes	0.9	0.88		0.23 (.25) ^†^	Removed				Item − 15
40	0.81	0.73		4.22 (.42) ^‡^	Removed				0.97	0.92		2.95 (.34) ^‡^	Removed				
41	1.24	1.27		1.12 (.29)				Yes	1.07	1.05		0.04 (.26) ^†^	Removed				Item − 16
42	0.8	0.58		6.24 (.58) ^‡^	Removed	anchor item			0.88	0.82		3.42 (.36) ^‡^	Removed				
43	0.93	0.85		2.34 (.27) ^‡^	Removed				0.79	0.64		3.07 (.37) ^‡^	Removed				
44	0.87	0.73		5.24 (.51) ^‡^	Removed				0.76	0.66		2.90 (.33) ^‡^	Removed				
45	0.88	0.77		6.49 (.60) ^‡^	Removed				0.99	0.95		2.30 (.28) ^‡^	Removed				
46	0.99	0.78		6.42 (.66) ^‡^	Removed	anchor item			0.97	0.71		1.14 (.11)		anchor item		Yes	Item − 17
47	0.94	0.77		5.65 (.55) ^‡^	Removed	anchor item			1.03	0.97		4.95 (.63) ^‡^	Removed	anchor item			
48	0.88	0.85		2.62 (.34) ^‡^	Removed				1.04	1.08		1.56 (.27) ^‡^	Removed				
49	1.07	1.06		5.10 (.50) ^‡^	Removed				1.08	0.92		2.95(.31) ^‡^	Removed				
50	0.83	0.74		2.55 (.27) ^‡^	Removed				0.91	0.76		3.18 (.41) ^‡^	Removed				
51	0.83	0.75		4.40 (.46) ^‡^	Removed				0.89	0.78		2.50 (.34) ^‡^	Removed				
52	0.92	0.74		6.82 (.75) ^‡^	Removed	anchor item			1.01	0.72		4.85 (.50) ^‡^	Removed	anchor item			
53	0.82	0.67		5.10 (.55) ^‡^	Removed				0.97	0.89		3.51 (.38) ^‡^	Removed				
54	0.77	0.63		2.91 (.32) ^‡^	Removed				0.83	0.75		2.46 (.30) ^‡^	Removed				

*Notes* U: Uncertainty, S: Stress

### DIF assessment for the brief version

Using the MIMIC approach, the DIF test identifies unfair items based on different respondent groups. The results of DIF testing indicate that the 3-item (Causes of this high-risk pregnancy), 26-item (Whether the high-risk pregnancy condition caused my baby’s death), and 29-item (Whether any change in high-risk pregnancy condition affects relationships within my family), that were maternal age reported differently. The 34-item (whether the choice of treatment is correct) showed significant inequivalence between primiparous and multiparous were deleted (Table 2). Therefore, it was removed from the original version. Thus, no DIF items across primiparas, multiparas, and maternal age appear in the brief version of the scale.

**Table 2:** DIF assessment with the USS-HRPV (*N* = 200)

Item	Uncertainty_Age			Uncertainty_Parity			Stress_Age			Stress_Parity		
	Estimate	*p*	Note	Estimate	*p*	Note	Estimate	*p*	Note	Estimate	*p*	Note
1	-	-	Anchor item	-0.25	0.185		0.11	0.182		-	-	Anchor item
2	0.04	0.796		0.03	0.867		0.03	0.753		-0.09	0.279	
3	-0.52	0.008	Prefers under 35 years old	0.09	0.670		0.18	0.043	Prefers older than 35 years old	0.04	0.657	
4	-0.16	0.213		-0.13	0.310		0.06	0.464		-0.03	0.729	
5	-0.09	0.425		0.02	0.848		0.12	0.125		-0.02	0.763	
6	-0.11	0.407		-0.09	0.494		0.01	0.926		-0.05	0.506	
7	0.08	0.324		-	-	Anchor item	0.09	0.203		-0.06	0.388	
8	0.06	0.538		-0.10	0.299		0.00	0.998		-0.04	0.594	
9	0.11	0.412		0.15	0.262		0.04	0.619		0.10	0.201	
10	-0.05	0.691		0.01	0.952		0.08	0.313		0.00	0.974	
11	0.02	0.884		-0.16	0.187		-0.01	0.942		-0.07	0.393	
12	-0.09	0.423		-0.05	0.627		-0.09	0.203		-0.07	0.347	
13	-0.03	0.803		-0.12	0.273		-0.06	0.432		-0.09	0.219	
14	-0.17	0.300		-0.28	0.097		-0.10	0.220		-0.11	0.161	
15	-0.11	0.526		-0.15	0.381		0.00	0.977		-0.12	0.144	
16	-0.10	0.629		-0.03	0.893		-0.02	0.865		0.01	0.876	
17	-0.15	0.340		-0.22	0.178		0.12	0.130		-0.07	0.384	
18	0.27	0.053		-0.05	0.746		0.15	0.056		-0.04	0.586	
19	-0.06	0.668		0.02	0.904		-0.01	0.884		0.02	0.843	
20	0.04	0.759		-0.04	0.743		0.09	0.250		0.09	0.212	
21	-0.03	0.766		-0.04	0.664		-0.04	0.566		0.03	0.713	
22	0.06	0.629		-0.08	0.519		0.05	0.484		0.06	0.449	
23	-0.01	0.891		0.11	0.207		-0.09	0.194		-0.03	0.674	
24	0.07	0.417		-	-	Anchor item	-0.08	0.249		-0.05	0.493	
25	0.06	0.417		-0.02	0.750		-0.06	0.314		-	-	Anchor item
26	0.06	0.677		0.10	0.475		0.20	0.022	Prefers older than 35 years old	0.06	0.470	
27	-0.02	0.910		0.02	0.886		0.13	0.116		0.10	0.254	
28	0.02	0.869		-0.03	0.808		0.05	0.514		0.00	0.976	
29	0.27	0.032	Prefers older than 35 years old	0.12	0.348		0.22	0.010	Prefers older than 35 years old	-0.04	0.630	
30	0.22	0.100		0.07	0.586		-0.04	0.534		-0.12	0.065	
31	0.14	0.289		0.16	0.219		0.10	0.244		0.00	0.989	
32	0.06	0.502		-0.04	0.707		0.00	0.985		-0.07	0.306	
33	-0.09	0.327		0.09	0.325		-0.05	0.500		0.03	0.691	
34	-0.08	0.514		0.00	1.000		-	-	Anchor item	-	-	Prefers primipara
35	0.06	0.686		0.01	0.970		-0.06	0.516		0.01	0.953	
36	0.05	0.741		0.21	0.126		0.01	0.935		0.07	0.422	
37	0.05	0.708		-0.03	0.834		-0.06	0.468		0.04	0.637	
38	-0.12	0.501		-0.01	0.963		0.08	0.328		-0.03	0.734	
39	0.20	0.194		0.12	0.444		0.12	0.162		-0.01	0.874	
40	-0.14	0.248		-0.03	0.774		-0.03	0.698		-0.07	0.364	
41	0.02	0.902		0.13	0.442		0.04	0.616		0.09	0.256	
42	-	-	Anchor item	0.02	0.835		0.00	0.965		-0.02	0.782	
43	0.06	0.540		0.05	0.624		0.04	0.533		0.03	0.618	
44	0.12	0.261		-0.12	0.258		0.00	0.955		-0.01	0.920	
45	0.14	0.125		0.05	0.588		0.00	0.985		0.01	0.942	
46	-	-	Anchor item	-	-	Anchor item	-	-	Anchor item	-	-	Anchor item
47	-	-	Anchor item	0.14	0.140		-	-	Anchor item	0.09	0.134	
48	-0.23	0.138		0.04	0.824		0.03	0.741		0.07	0.406	
49	0.13	0.240		0.02	0.881		0.05	0.566		0.08	0.307	
50	0.01	0.928		-0.06	0.533		0.07	0.309		0.04	0.596	
51	-0.12	0.280		-0.04	0.700		0.04	0.578		0.07	0.342	
52	-0.09	0.266		-	-	Anchor item	-	-	Anchor item	-	-	Anchor item
53	0.01	0.945		0.05	0.614		0.06	0.384		0.04	0.545	
54	0.10	0.251		0.04	0.674		0.06	0.452		0.06	0.404	

*Notes* Anchor items were identified in the first step of the DIF-Free-then-DIF (DFTD) strate

As such, this study constructed the short form of the USS-HRPV-C: four items in the uncertainty subscale and nine items in the stress subscale. To ensure the brief version retained the psychometric properties of the original scale, we calculated the correlation of item difficulty estimates between the original and brief versions. The Pearson correlation coefficient for item difficulty was *r* = 1.0 (*p* < 0.001) for both the uncertainty and stress subscales. The results indicate that the items in the original and brief versions have similar psychometric properties.

### Panel discussion

We determined the final USS-HRPV-C brief version based on expert panel discussion with literature support. Previous research indicates that the principal causes of fetus death were complications of High-Risk Pregnancy [60, 61]. Women facing high-risk pregnancies often experience fears and concerns related to their health and the fetus’s health [62]. In this regard, five items that have relatively poor item statistics were retained in the scale through the panel discussion: Item-14 (Whether I will go smoothly from pregnancy to delivery), Item 15 (Whether the baby will be safe and healthy), Item 23 (Whether delays in treatment will influence the baby), Item-26 (Whether the high-risk pregnancy condition causes my baby death), Item-27 (Whether the high-risk pregnancy condition causes my death), and Item-37 (My ability to handle emotions related to the pregnancy condition). Although DIF appeared in item-26, based on the literature, the panel decided to retain items 14, 15, 26, 27, and 37, as shown in Table 1.

### Validity and reliability of the brief version of the USS-HRPV-C

Eventually, a 17-item brief form of USS-HRPV-C was constructed where items 2, 11, 39, and 41 were retained in the uncertainty subscale. Items 4, 5,10, 11, 17, 20, 22, 35, and 46 in the stress subscale (Table 1).

The findings of the CFA in the current study indicated that all data were satisfactory and acceptable in terms of the data-model fit indices for the uncertainty and stress of the brief version, the result including the 1.33–2.63 for χ2 /df, 0.980 – >0.983 for NNFI, 0.980 – 0.985 for CFI, 0.041 – 0.091 for RMSEA, and 0.081 – 0.096 for SRMR as shown in Table 3.

**Table 3:** Confirmatory factor analysis(CFA) result in original and brief scale of USS-HRPV-C

Fit indices	Original scale		Brief scale	
	Uncertainty	Stress	Uncertainty	Stress
χ^2^	1827.79	2936.95	40.78	202.51
Df	1377	1377	27	77
χ^2^/df	1.33	2.13	1.51	2.63
NNFI	0.980	0.983	0.980	0.983
CFI	0.980	0.983	0.985	0.983
RMSEA	0.041	0.075	0.051	0.091
SRMR	0.089	0.096	0.083	0.081

*Notes* NNFI = non-normed fit index; CFI = comparative fit index; RMSEA = root mean square of error approximation; SRMR = standardized root mean square residual

Regarding to concurrent validity, we compared the brief version of USS-HRPV-C score with the VAS two subscales. The uncertainty subscale of the USS-HRPV-C was significantly positive correlated with the overall uncertainty of VAS (*r* = 0.47, *p* < 0.001); the stress subscale was also significantly positive correlated with the overall stress of VAS (*r* = 0.68, *p* < 0.001). Futhremore, the 54-item and 17-item were significantly positive correlated in uncertainty subscale (*r* = 0.937, *p* < 0.001) and stress subscale ( *r* = 0.941, *p* < 0.001).

The composite reliability (CR) of the uncertainty subscales of the 54-item and 17-item were 0.955 and 0.856, the stress subscales were 0.969 and 0.910. The average variance extracted (AVE) of the uncertainty subscales were 0.464 and 0.410, the stress subscales were 0.544 and 0.578, respectively. The average variance extracted (AVE) values for the uncertainty and stress constructs in both the original and brief scales were below the recommended threshold of 0.50, ranging from 0.410 to 0.578. However, according to Fornell and Larcker [63], AVE values below 0.50 can be acceptable if they have high composite reliability (CR). Our study’s CR values were strong, ranging from 0.856 to 0.969, indicating sufficient internal consistency. Therefore, despite the lower AVE values, the constructs demonstrate adequate convergence. This reflects convergence and discriminant validity, as shown in Table 4.

**Table 4:** Convergence and discriminant validity in original and brief scale of USS-HRPV-C

Fit indices	Original scale (54 items)		Brief scale (17 items)	
	Uncertainty	Stress	Uncertainty	Stress
CR	0.955	0.969	0.856	0.910
AVE	0.464	0.544	0.410	0.578

Notes CR = composite reliability; AVE = average variance extracted

The internal consistency of the brief version has a Cronbach’s alpha of 0.90 in the uncertainty subscale and a Cronbach’s alpha of 0.92 in the stress subscale. The 17-item brief version of the USS-HRPV-C has satisfied reliability.

In this study, we conducted analyses for the short form by separating it into two dimensions: uncertainty and stress. Ultimately, a 17-item brief version was developed, comprising four items from the uncertainty subscale, nine items from the stress subscale, and an additional four items selected through expert panel review (Table 1).

## Discussion

This study proposed a shortened 17-item version of the USS-HRPV-C and the procedures for item deletion and retention were described in detail. This study applied the Rasch analysis to offer the testing of efficiency, strengthens item reduction methodology, and allows for the conjoint measurement of persons and items on the same dimension. By removing 37 items from the original 54-item USS-HRPV-C version, the 17-item brief version retains the similar psychometric properties as its original version. This evidence supports that the brief form of the USS-HRPV-C can measure the experience of uncertainty and stress in women with high-risk pregnancies.

The findings of this study suggest that five items (i.e., Item 14, 15, 26, 27, and 37) are related to the pregnancy process, fetal and maternal health, and delivery outcomes. This is consistent with the findings reported by Sheen and Slade [64], which suggest that uncertainty underlies women’s general and specific concerns regarding childbirth. In addition, nine items (i.e., Item 2, 4, 5, 10, 11, 17, 20, 22, and 41) in the brief version of USS-HRPV-C involve discomfort symptoms concerned by pregnant women. These items are consistent with the findings of Lee, Ayers [65], indicating that complications due to high-risk pregnancy increase mothers’ uncertainty towards pregnancy outcomes. High-risk pregnant women struggle to identify the exact causes of their pregnancy complications and physical symptoms [66]. Two items (i.e., Item 37 and 39) are related to coping strategies of uncertain psychological conditions. Previous studies reported uncertainty is associated with high levels of perceived stress and psychological distress [67, 68]. Item 46 is related to the health caregivers, confirming the association between care providers and high-risk pregnancy uncertainty [64].

Uncertainty may be associated with the surrounding disease itself, treatment, childbirth, and neonatal outcomes [69]. Uncertainty is a stressful condition that affected the health of the individual [12]. Carter, Tribe [70] proposed the significant nature of high risk pregnancy (e.g. preterm labour) is uncertainty. Additionally, Çevik and Yağmur [13] points to that psychological well-being decreased when pregnant women experience uncertainty. Therefore, a brief version of USS-HRPV-C is affordable and efficient for pregnant women to evaluate their stress related to pregnancy.

The brief version of USS-HRPV-C offers several significant advantages. The brief version only contains one-third the number of items of the original USS-HRPV-C. The brief version may reduce the respondents’ burdens to fill out the scale. Data quality, completeness, and reliability may also be enhanced, and the respondents may be more willing to participate in studies. This study was the first to use the Rasch model to assess the USS-HRPV-C brief version’s psychometric properties and improve testing efficiency.

The current study successfully identified the 17 items to include in the brief version of the USS-HRPV-C scale while maintaining the satisfied psychometric properties. The proposed 17-item scale can be beneficial in assessing uncertainty and stress levels among high-risk pregnant women in clinical practice. However, the brief version of the USS-HRPV-C is needed to be further examined to validate its usefulness in clinical settings. A cross-cultural validity study across different languages and countries is necessary for future studies.

### Limitations and suggestions

This USS-HRPV-C brief version was tested on high-risk pregnant women and cannot be applied to women with low-risk pregnancies. Using the brief version in Western countries would consider whether the measure is culturally appropriate. Additionally, more than three-quarters of our participants had a university education, which may introduce bias and limit the generalizability of our findings. Future research should test the USS-HRPV-C among high-risk pregnant women with lower educational attainment (e.g., those without a university degree) to determine its applicability across diverse educational backgrounds.

Some items in the brief version are repetitive, differing only in their wording concerning uncertainty and stress. Future research is recommended to explore the relationship between uncertainty and stress in women with high-risk pregnancies.

We also acknowledge this study’s following limitations. First, the small size and convenience sampling nature of the sample. Due to data collection constraints during the COVID-19 pandemic and the inherent challenges of recruiting participants from the high-risk pregnancy population, it was difficult to collect additional data for the brief version. In this study, we developed the 17-item brief version based on the original 54-item scale to provide a preliminary foundation for future validation. Future research will focus on collecting independent and larger samples to test further and validate the short version, as we recognize the importance of independent data in confirming their psychometric properties.

## Conclusion

The 17-item brief version of the USS-HRPV-C scale demonstrated satisfactory psychometric properties after the Rasch analysis, allowing the scale to assess uncertainty and stress among high-risk pregnancies while maintaining minimal burden on the respondents. The brief version of the USS-HRPV-C provides clinical professionals with a quick and accurate assessment tool by reducing the number of items that adequately reflect the psychological degree of uncertainty and stress associated with high-risk pregnancies.

## Electronic supplementary material

Below is the link to the electronic supplementary material.

Supplementary Material 1

## Data Availability

Availability of data and materials are available upon request from the corresponding author.

## Abbreviations

USS-HRPV: Uncertainty Stress Scale-High Risk Pregnancy Version
CFA: Confirmatory Factor Analysis
DIF: Differential Item Functioning
TAM: Test Analysis Modules
IUFD: Intrauterine Fetal Death
GDM: Gestational Diabetes Mellitus
HDP: Hypertensive Disorders of Pregnancy
OPD: Outpatient Department
lavaan: Latent variable analysis
DWLS: Diagonally Weighted Least Squares
ML: Maximum Likelihood
MLR: Maximum Likelihood Robust
NNFI: Non-normed Fit Index
TLI: Tucker-Lewis Index
RMSEA: Root-Mean-Squared Error of Approximation
SRMR: Standardized root-mean-squared Residual
MPCM: Multidimensional Partial Credit Model
PCM: Partial Credit Model
RSM: Rating Scale Model
MNSQ: Mean Square
DIF: Differential Item Functioning
DFTD: DIF-Free-Then-DIF
SIBTEST: Simultaneous Item Bias Test
MIMIC: Multiple Indicators and Multiple Causes
SEM: Structural Equation Modeling
CR: Composite Reliability
AVE: Average Variance Extracted
