# Supplementary material for: Validation of the uncertainty stress scale-high-risk pregnancy - Chinese brief version: Rasch analysis
Source: BMC Pregnancy Childbirth. 2025 Jan 6;25:6. doi: 10.1186/s12884-024-07078-7 (PMC11702129; doi:10.1186/s12884-024-07078-7)
Supplement: Supplementary file 1 — Supplementary Material 1 [file 12884_2024_7078_MOESM1_ESM.docx]

Additional file 1

Scree plot of the uncertainty subscale

Scree plot of the stress subscale

Additional file 2

Item response mean (SD), skewness and kurtosis values, inter-item correlations, and factor loadings with the USS-HRPV (N = 200)

| **USS-HRPV-C item description** | **Subscale** | **Mean (SD)** | **Skewness** | **Kurtosis** | **Item-total correlation** |
| --- | --- | --- | --- | --- | --- |
| 1. Whether physical changes can be detected early | U | 2.23 (1.31) | .68 | -.72 | .08 |
|  | S | 1.60 (.61) | .51 | -.62 | .33 |
| 1. Is the pregnancy condition stable | U | 2.32 (1.13) | .34 | -.74 | .49 |
|  | S | 1.86 (.62) | .10 | -.46 | .47 |
| 1. Causes of this high-risk pregnancy | U | 2.30 (1.43) | .68 | -.93 | .29 |
|  | S | 2.03 (.65) | -.03 | -.65 | .45 |
| 1. Whether to continue to maintain the present level of fenclonine | U | 1.78 (.98) | 1.16 | .87 | .58 |
|  | S | 1.69 (.60) | .28 | -.62 | .58 |
| 1. Present state of pregnancy condition | U | 1.61 (.89) | 1.41 | 1.24 | .65 |
|  | S | 1.71 (.58) | .15 | -.56 | .59 |
| 1. What questions of pregnancy to ask medical specialists | U | 1.67 (.94) | 1.24 | .72 | .48 |
|  | S | 1.37 (.52) | .96 | -.22 | .57 |
| 1. Whether changing lifestyle will help pregnancy condition | U | 1.27 (.61) | 2.76 | 9.17 | .42 |
|  | S | 1.34 (.52) | 1.22 | .49 | .40 |
| 1. How to make sense of my doctor telling me pregnant situation | U | 1.38 (.69) | 2.14 | 4.97 | .56 |
|  | S | 1.32 (.48) | 1.03 | -.30 | .50 |
| 1. How does the effectiveness of high-risk pregnancy treatments | U | 1.73 (.97) | 1.25 | .90 | .53 |
|  | S | 1.53 (.58) | .59 | -.60 | .54 |
| 1. Whether the condition of high-risk pregnancy is under control | U | 1.93 (.99) | .85 | .05 | .56 |
|  | S | 1.68 (.55) | .48 | -.65 | .55 |
| 1. Whether the pregnancy condition changes you be able to feel the symptoms | U | 1.71 (.90) | 1.06 | .10 | .59 |
|  | S | 1.64 (.60) | .35 | -.66 | .57 |
| **USS-HRPV-C item description** | **Subscale** | **Mean (SD)** | **Skewness** | **Kurtosis** | **Item-total correlation** |
| 1. How to talk to someone about a high-risk pregnancy situation | U | 1.47 (.82) | 1.69 | 2.22 | .57 |
|  | S | 1.36 (.54) | 1.21 | .51 | .56 |
| 1. How to adapt to various explanations given by others about the pregnancy situation | U | 1.58 (.84) | 1.50 | 1.84 | .60 |
|  | S | 1.43 (.57) | .93 | -.12 | .53 |
| 1. Whether I will go smoothly from pregnancy to delivery | U | 2.57 (1.25) | .52 | -.59 | .58 |
|  | S | 2.06 (.60) | -.02 | -.26 | .49 |
| 1. Whether the baby will be safe and healthy | U | 2.51 (1.24) | .66 | -.44 | .57 |
|  | S | 2.09 (.61) | -.05 | -.36 | .50 |
| 1. Whether the next pregnancy will be the same as this one | U | 3.08 (1.43) | -.04 | -1.26 | .37 |
|  | S | 1.82 (.66) | .21 | -.75 | .40 |
| 1. Whether symptoms can be controlled | U | 2.57 (1.25) | .35 | -.63 | .62 |
|  | S | 1.88 (.59) | .03 | -.22 | .63 |
| 1. Whether this high-risk pregnancy affected my usual activities | U | 1.80 (1.04) | 1.38 | 1.43 | .39 |
|  | S | 1.75 (.58) | .09 | -.45 | .52 |
| 19. Whether the doctor can manage my disease | U | 1.67 (1.02) | 1.78 | 2.81 | .56 |
|  | S | 1.47 (.58) | .80 | -.32 | .64 |
| 1. How to manage my symptoms such as bleeding, uterine contractions | U | 1.71 (.90) | 1.15 | .83 | .66 |
|  | S | 1.68 (.55) | .06 | -.66 | .54 |
| 1. Whether the choice of treatment is correct | U | 1.39 (.71) | 2.04 | 3.95 | .60 |
|  | S | 1.42 (.52) | .64 | -.90 | .63 |
| 1. Whether symptoms return | U | 1.73 (.92) | 1.48 | 2.16 | .45 |
|  | S | 1.66 (.58) | .21 | -.66 | .55 |
| 1. Whether follow-up treatment was appropriate | U | 1.36 (.67) | 2.02 | 3.92 | .55 |
|  | S | 1.41 (.51) | .61 | -1.10 | .57 |
| 1. My understanding of the treatment I have received and am receiving | U | 1.31 (.61) | 2.51 | 8.35 | .50 |
|  | S | 1.33 (.50) | 1.11 | .03 | .54 |
| **USS-HRPV-C item description** | **Subscale** | **Mean (SD)** | **Skewness** | **Kurtosis** | **Item-total correlation** |
| 1. How to communicate with healthcare providers about my care | U | 1.33 (.59) | 1.80 | 2.84 | .57 |
|  | S | 1.22 (.42) | 1.59 | 1.13 | .49 |
| 1. Whether the high-risk pregnancy condition caused my baby`s death | U | 2.07 (1.07) | .92 | .34 | .55 |
|  | S | 1.86 (.64) | .13 | -.60 | .53 |
| 1. Whether the high-risk pregnancy condition causes my death | U | 2.00 (1.09) | 1.05 | .46 | .57 |
|  | S | 1.74 (.62) | .26 | -.63 | .46 |
| 1. Whether the treatment received reduces the high- risk of pregnancy condition | U | 1.58 (.95) | 1.88 | 3.39 | .53 |
|  | S | 1.50 (.57) | .63 | -.57 | .55 |
| 1. Whether any change in high-risk pregnancy condition affects relationships within my family | U | 1.57 (.92) | 1.80 | 2.96 | .38 |
|  | S | 1.48 (.63) | .97 | -.11 | .41 |
| 1. Whether any change in high-risk pregnancy condition affects relationships with my colleagues or friends | U | 1.57 (.98) | 1.96 | 3.57 | .28 |
|  | S | 1.25 (.48) | 1.66 | 1.89 | .40 |
| 1. Whether pregnancy condition affects my life goals | U | 1.60 (.94) | 1.82 | 3.28 | .29 |
|  | S | 1.48 (.61) | .91 | -.17 | .47 |
| 1. Whether what I am doing about my condition help me | U | 1.41 (.71) | 1.76 | 2.52 | .49 |
|  | S | 1.43 (.54) | .75 | -.54 | .54 |
| 1. Whether I can depend on test results as an indicator of pregnancy condition | U | 1.44(.72) | 1.81 | -.37 | .59 |
|  | S | 1.39 (.52) | .88 | -.37 | .59 |
| 1. Whether my condition affects my sex life | U | 1.48 (.91) | 2.31 | 5.32 | .32 |
|  | S | 1.17 (.39) | 2.02 | 2.91 | .41 |
| 1. Whether delays in treatment affect the baby | U | 1.77 (1.09) | 1.38 | 1.08 | .54 |
|  | S | 1.70 (.65) | .40 | -.73 | .61 |
| 1. The seriousness of the pregnancy condition | U | 1.88(1.04) | 1.06 | .31 | .64 |
|  | S | 1.75 (.62) | .24 | -.62 | .69 |
| 1. My ability to handle emotions related to the pregnancy condition | U | 2.00 (1.08) | .91 | .12 | .64 |
|  | S | 1.76 (.62) | .23 | -.61 | .59 |
| **USS-HRPV-C item description** | **Subscale** | **Mean (SD)** | **Skewness** | **Kurtosis** | **Item-total correlation** |
| 1. When symptoms occur | U | 2.67 (1.32) | .25 | -1.00 | .56 |
|  | S | 1.87 (.62) | .10 | -.47 | .55 |
| 1. Whether I have difficulty coping with pregnancy condition | U | 2.32 (1.17) | .58 | -.50 | .62 |
|  | S | 1.85 (.64) | .15 | -.61 | .66 |
| 40. Whether the quality of the information received is correct | U | 1.64 (.92) | 1.65 | 2.64 | .59 |
|  | S | 1.42 (.55) | .88 | -.25 | .63 |
| 41. How long the symptoms last | U | 2.27 (1.23) | .82 | -.19 | .47 |
|  | S | 1.81 (.60) | .11 | -.42 | .53 |
| 42. Whether family members or health care workers told the truth about pregnancy condition | U | 1.26 (.69) | 3.20 | 11.23 | .51 |
|  | S | 1.36 (.53) | 1.11 | .20 | .53 |
| 43. Whether I would choose to receive all the treatments recommended | U | 1.38 (.70) | 2.10 | 4.29 | .44 |
|  | S | 1.37 (.52) | .96 | -.22 | .63 |
| 44. What abnormal symptoms mean in terms of pregnancy condition | U | 1.45 (.78) | 2.06 | 4.63 | .58 |
|  | S | 1.42 (.56) | .94 | -.11 | .64 |
| 45. Whether they might find something wrong when I go for an ultrasound | U | 1.32 (.70) | 2.93 | 10.35 | .51 |
|  | S | 1.48 (.63) | .99 | -.08 | .55 |
| 46. Whether I will be well cared for by the nurses | U | 1.27 (.65) | 2.79 | 8.48 | .43 |
|  | S | 1.18 (.38) | 1.67 | .85 | .40 |
| 47. Whether I will be well cared for by health professionals other than nurses | U | 1.38 (.74) | 2.43 | 6.75 | .49 |
|  | S | 1.24 (.44) | 1.40 | .47 | .46 |
| 48. The cause of my symptoms | U | 2.03 (1.19) | .94 | -.10 | .61 |
|  | S | 1.59 (.64) | .64 | -.56 | .56 |
| 49. Whether I can depend on people who are important to me to be there when I need them | U | 1.49 (.85) | 1.78 | 2.81 | .48 |
|  | S | 1.39 (.58) | 1.20 | .46 | .52 |
| 50. How to know what symptoms I should be aware of | U | 1.52(.75) | 1.41 | 1.50 | .59 |
|  | S | 1.43 (.52) | .62 | -.93 | .57 |
| **USS-HRPV-C item description** | **Subscale** | **Mean (SD)** | **Skewness** | **Kurtosis** | **Item-total correlation** |
| 51. How to choose the treatments | U | 1.24 (.57) | 3.13 | 12.40 | .58 |
|  | S | 1.49 (.55) | .58 | -.71 | .56 |
| 52. Whether by following the treatment plan recommended to me will help | U | 1.21 (.43) | 1.80 | 2.24 | .44 |
|  | S | 1.21 (.43) | 1.80 | 2.24 | .45 |
| 53. What to look for to check the state of my high-risk pregnancy situation | U | 1.51 (.82) | 1.62 | 2.15 | .60 |
|  | S | 1.36 (.52) | 1.03 | -.04 | .54 |
| 54. Whether the treatment I will be having reduces the risk of my pregnancy | U | 1.47 (.78) | 1.36 | 1.03 | .65 |
|  | S | 1.47 (.59) | .87 | -.22 | .62 |

*Notes.* Subscale U: Uncertainty, S: Stress

Additional file 3-1


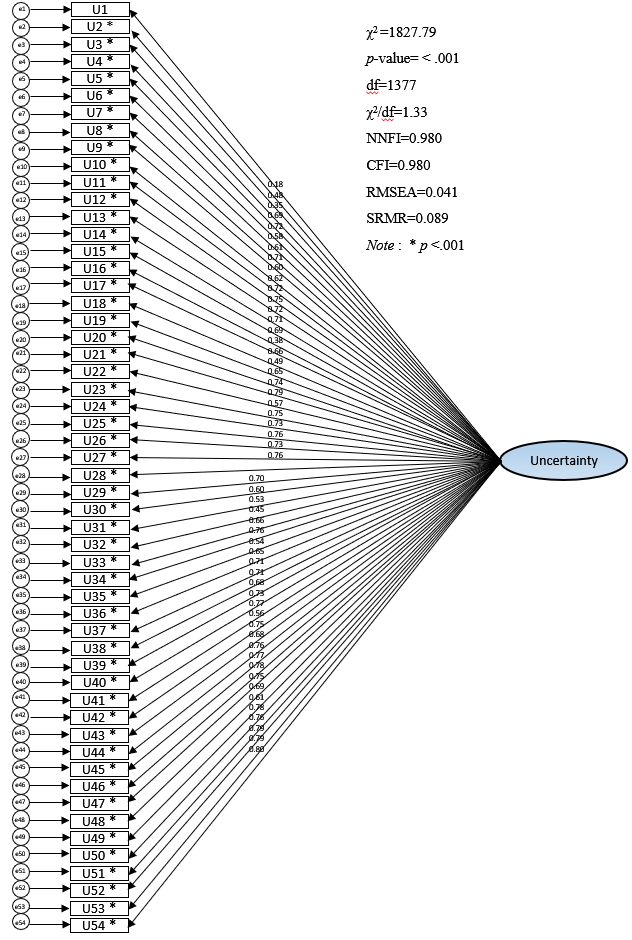


Confirmatory factor analysis of the 54-item uncertainty subscales of the USS-HRPV

Additional file 3-2


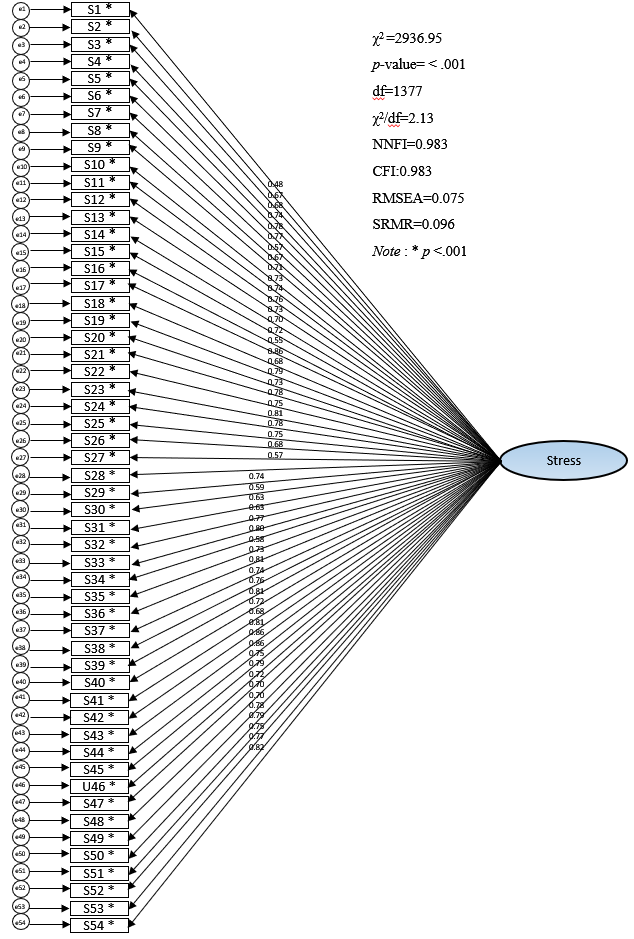


Confirmatory factor analysis of the 54-item stress subscales of the USS-HRPV
